# P15 What is the value of direct-from-blood culture ASTs in the study of bacterial susceptibility to antibiotics during bacteraemia?

**DOI:** 10.1093/jacamr/dlac053.015

**Published:** 2022-05-31

**Authors:** Samar Akbi, Nassim Ahmed, Nadjet Aggoune, Ali Zerouki

**Affiliations:** Mohamed Seghir Nekkache University Hospital, Algiers, Algeria

## Abstract

**Objectives:**

To evaluate the results obtained with direct-from-blood culture antimicrobial susceptibility tests (ASTs) for the study of bacterial susceptibility to antibiotics during bacteraemia.

**Material and methods:**

This was a prospective study in which 124 direct ASTs were performed directly from positive blood cultures according to the CLSI technique using Mueller–Hinton CHROMagar Orientation medium. Twenty-one antibiotics were tested. The resulting diameters were read after 8 h and 18 h of incubation and interpreted according to the CLSI breakpoints. Thus, a standard AST was performed and interpreted for all isolated strains according to CLSI recommendations. The results were analysed by calculating the rates of categorical agreements (CA) and disagreements represented by minor errors (mE), major errors (ME) and very major errors (VME).

**Results:**

One hundred and twenty-four strains were isolated, including *Escherichia coli* (26; 20.97%), *Klebsiella pneumoniae* (25; 20.16%) and SCN (21; 16.94%). More than half of the isolates (69; 55.65%) were MDR bacteria. It was found that the overall percentage CA (%CA) obtained at 18 h (94.43%) was higher than the one obtained at 8 h (87.32%). Disagreement rates obtained at 8 h and 18 h remain acceptable. The best %CA was obtained with non-fermenting GNB (98.74%) and the lowest with *Staphylococcus* species (90.70%). For Enterobacteriaceae, an excellent overall %CA (94.32%) was found at 18 h with 96.3% for CTX, 95.38% for ETP, 95.31% for CRO and 92.19% for MEM. Lower rates were noted with IPM (88.71%), CAZ (87.5%) and CIP (85.71%); due to mE for CAZ (12.5%) and mE and ME for IPM (9.68% mE; 1.85% ME) and CIP (12.7% mE; 3.23% ME). Particular attention should be given to clindamycin and teicoplanin tested with *Staphylococcus* species as they record low categorical agreements at 8 h and 18 h.

**Conclusions:**

The encouraging results obtained at 18 h suggest a possible future implementation of the direct-from-blood culture AST as a routine technique for studying susceptibility to antibiotics during bacteraemia. However, the standard AST remains the reference technique.

